# Orthodontic-Surgical Correction of a Skeletal Class III With Severe Facial Asymmetry Treated With Double Jaw Surgery and Mandibular Shaving: A Case Report

**DOI:** 10.7759/cureus.98899

**Published:** 2025-12-10

**Authors:** Gerardo Martínez-Suárez, Luis Pablo Cruz-Hervert, Martha Elizabeth Tovar-Martínez, Angelica Julián-Castrejón, José Rubén Gómez-Garibo, Juan Antonio Maldonado-Moreno

**Affiliations:** 1 Dentistry, Clínica Odontológica Aragón, Facultad de Estudios Superiores Iztacala, Universidad Nacional Autónoma de México, Tlalnepantla de Baz, MEX; 2 Medicine, División de Estudios de Posgrado e Investigación (DEPeI) en Odontología, Universidad Nacional Autónoma de México, Ciudad de México, MEX; 3 Dentistry, El Hospital Regional de Alta Especialidad de Ixtapaluca (HRAEI), Ixtapaluca, MEX; 4 Nuclear Medicine, Hospital Regional de Alta Especialidad de Ixtapaluca, Ixtapaluca, MEX; 5 Orthodontics, Hospital Regional de Alta Especialidad de Ixtapaluca, Ixtapaluca, MEX

**Keywords:** bimaxillary surgery, digital planning, facial asymmetry, “orthognathic surgery”, skeletal class iii deformities

## Abstract

A 17-year-old female with severe facial asymmetry and skeletal Class III malocclusion, whose skeletal growth was already complete, presented with left-sided mandibular prognathism, a 5 mm chin height discrepancy (higher on the right side), and a 7.6 mm chin deviation to the left, together with paranasal depression and a 4 mm cant of the maxillary occlusal plane, positioned higher on the right side.

The patient exhibited significant dental asymmetry, including a Class III molar relationship (7 mm) and canine relationship on the right side and a Class II molar relationship (end-on) on the left, along with a left posterior crossbite. After evaluation by the oral and maxillofacial surgery team, it was determined that she required bimaxillary orthognathic surgery combined with chin border reduction.

The treatment consisted of bimaxillary surgery and inferior chin border shaving, including a Le Fort I osteotomy for 5.5 mm of maxillary advancement, asymmetric intrusion (2 mm right-sided downward repositioning and 2 mm left-sided intrusion), and bilateral mandibular ramus osteotomies to achieve a 5.5° rotation to the right and counterclockwise mandibular rotation. The final positioning was adjusted according to the preoperative surgical plan, with careful verification of occlusal alignment, overjet, overbite, facial symmetry, and lip commissure level before rigid fixation was performed.

## Introduction

Due to the complexity of diagnosis and treatment planning, patients with facial asymmetry-particularly those with skeletal Class III malocclusion-present a significant therapeutic challenge. Class III malocclusion combined with facial asymmetry is a multifactorial dentofacial deformity that results in both functional limitations and aesthetic concerns. In adult patients, where orthopedic growth modification is no longer an option, this condition typically requires a comprehensive treatment approach. The integration of orthodontic treatment with orthognathic surgery has proven to be effective and is widely accepted for achieving harmony between the skeletal, dental, and soft tissue components of the face [1-3].

The prevalence of facial asymmetry varies widely across populations and facial regions, with global estimates ranging from 21% to 85% [4]. The lower third of the face is most commonly affected, showing asymmetry in approximately 74% of cases, followed by the midface (36%) and upper third (5%) [5]. The most common cause is bilateral mandibular asymmetry, which is associated with facial asymmetry in nearly 50% of patients with mandibular prognathism [6,7].

Patients with skeletal asymmetry also exhibit soft tissue asymmetry during dynamic expressions, such as smiling. The range of movement tends to be greater on the non-deviated side than on the deviated side [8]. Although combined orthodontic and orthognathic treatment significantly improves facial symmetry, some degree of asymmetry may persist postoperatively [9]. In cases where skeletal asymmetry involves the maxillomandibular complex-particularly in the presence of a pronounced occlusal plane cant. For patients with a pronounced occlusal plane cant, Le Fort I osteotomy can be particularly beneficial. The procedure allows for precise repositioning of the maxilla, which is crucial for correcting the occlusal plane and achieving a balanced bite [10,11].

Bimaxillary orthognathic surgery can nearly correct facial asymmetry, aligning facial features with the principles of the double-cross grid. Improvements typically include correction of occlusal plane inclination, dental midline alignment, chin deviation, and bilateral gonial width symmetry [9,12].

Correction of skeletal Class III malocclusion is achieved by repositioning the jaws along the occlusal plane through anterior or posterior movements. Mandibular asymmetries can be addressed using various osteotomy techniques-including sagittal, vertical, and inverted “C” ramus osteotomies-to allow for tailored advancements, setbacks, or mixed modifications according to individual anatomical requirements [13].

The use of digital imaging and virtual surgical planning has greatly enhanced clinicians' ability to diagnose and treat facial skeletal discrepancies. These tools improve assessment of airway function, feeding, and speech and are instrumental in correcting facial asymmetries, managing occlusal plane discrepancies, and optimizing jaw positioning and skeletal alignment [14-16].

The objective of this report is to present the successful treatment of a 17-year-old female patient with skeletal Class III malocclusion and severe facial asymmetry, managed through double-jaw surgery, including Le Fort I osteotomy with asymmetric maxillary advancement and impaction and bilateral sagittal split osteotomy (BSSO).

## Case presentation

A 17-year-old girl with severe facial asymmetry and skeletal Class III malocclusion was referred to Hospital Regional de Alta Especialidad de Ixtapaluca (HRAEI). No pertinent pathological antecedents were found during the medical-dental history. An intraoral examination showed no symptoms of illness. The patient had an oval face with noticeable facial asymmetry toward the left side, according to photographic analysis. With a discordant grin arc with the lower lip, a distorted vertical exposure of the upper incisors when smiling, and an enlarged lower facial third, the face was disproportionate.

She had a straight profile, an obtuse nasolabial angle, a slight bimaxillary protrusion, a retrusive chin that was 6.5 mm to the left, unequal eye levels, and a dip in the midfacial third at the level of the malar bone. Intraoral analysis revealed a canted occlusal plane (OP), characterized by midlines that did not align with the facial midline. Specifically, the maxillary midline was 1.6 mm to the right, and the mandibular midline was 7.6 mm to the left. A Class III molar relationship on the right and Class II on the left, a Class III canine relationship on the right and Class II on the left, a left posterior crossbite, and severe upper and moderate lower dental crowding were all present.

**Figure 1:**
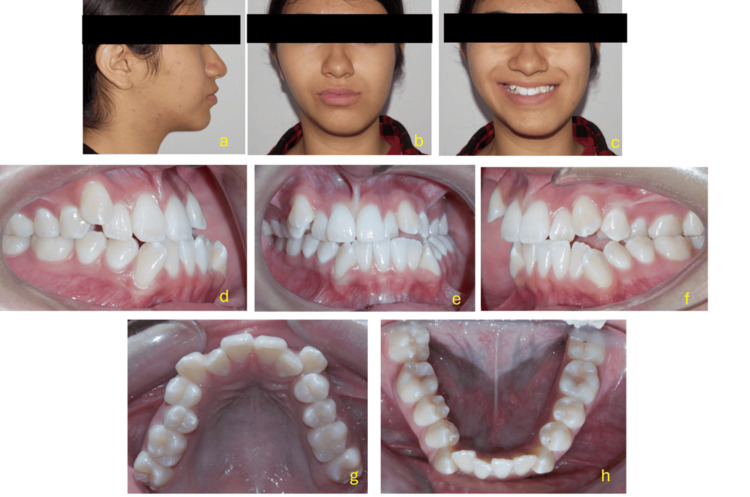
Extraoral and intraoral clinical evaluation The images display the patient’s extraoral and intraoral records at the pre-treatment stage. (a) Lateral view showing a convex profile with left mandibular protrusion and mild facial asymmetry, including chin deviation toward the left. (b) Frontal view at rest revealing facial asymmetry with a canted maxillary occlusal plane and deviation of the mandibular midline to the left. (c) Frontal smiling view where facial asymmetry remains evident, along with uneven lip commissure heights and asymmetric dental display. (d) Right intraoral lateral view demonstrating a Class III molar and canine relationship with a negative overjet on that side. (e) Frontal intraoral view revealing a transverse discrepancy characterized by a left posterior crossbite, severe maxillary crowding, moderate mandibular crowding, and deviation of the lower midline to the left. (f) Left intraoral lateral view showing a Class II molar (edge-to-edge) and canine relationship, with the upper left side positioned slightly higher. (g) Maxillary occlusal view illustrating severe crowding, irregular alignment of the incisors, and transverse constriction of the maxillary arch. (h) Mandibular occlusal view showing moderate crowding and slight asymmetry of the mandibular arch, with collapse in the left posterior region.

There was a left posterior crossbite and a canted occlusal plane, with the right side being 4 mm lower than the left. A minor maxillary collapse, palatal coronal inclination, lingual coronal inclination of the lower molars on the left side, and inadequate space for the upper canines were all identified by maxillary arch analysis. Asymmetric sagittal occlusion was also seen, with tooth 36 positioned distally and the lower left molars exhibiting severe lingual torque. All these characteristics pointed to a significant change in the occlusal plane. Additionally, the correlations between overjet and overbite were altered (Figure 1).

Models analysis

The plaster models illustrate a severe dental crowding in both arches, a bilateral posterior crossbite, an anterior open bite, and a Class III molar relationship. The occlusal views confirm arch form discrepancies and highlight significant transverse and sagittal imbalances, crucial for diagnosis and surgical-orthodontic planning.

**Figure 2:**
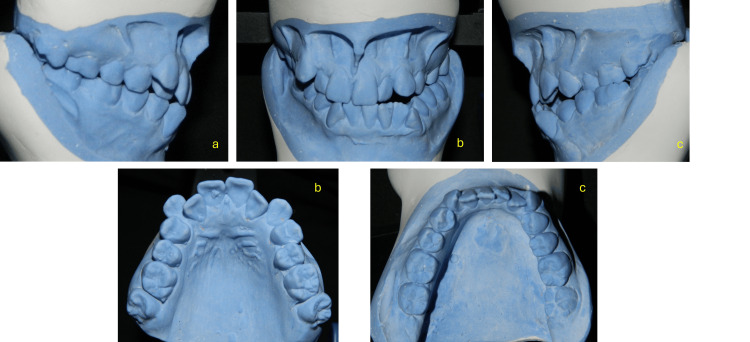
Pretreatment study models

In the functional analysis, we observe that maximum mouth opening was 43 mm. The patient exhibited no functional guidance; during protrusive and lateral movements, interferences were observed on both the working and balancing sides. Additionally, there was a premature contact point on the left side between teeth 27 and 37, which forced the mandible to deviate further to the left (Figure 2).

Sagittal diagnosis

**Figure 3:**
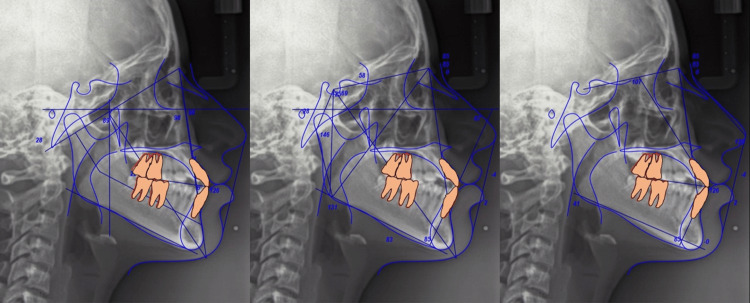
Description of the cephalometric characteristics (pre-treatment)

She presented with a Class III skeletal relationship (ANB -1.2°), maxillary retrusion, severe mandibular body overlength (83 mm), marked anteroposterior deficiency of the zygomatic buttress, and maxillary dentoalveolar proclination, and the mandibular incisors exhibited retroclination relative to their bony base. Her facial pattern was hyperdivergent vertically (Jarabak rotational value 401°) with a moderate brachyfacial growth direction (Ricketts). The maxillary incisors were slightly proclined, while the mandibular incisors were retroclined relative to the basal bone. Her molar and canine relationships were Class III on the right side and Class II on the left (Figure 3).

The details of the cephalometric changes are shown in Table 1. From the initial to the post-surgical assessment, the patient showed notable improvements in maxillomandibular relationships and dental inclinations. The SNA, SNB, and ANB angles increased, reflecting advancement of both the maxilla and mandible and improved sagittal jaw relationships. Maxillary and mandibular depths also increased, indicating forward positioning of both jaws. Lower facial height and facial axis increased, suggesting enhanced vertical proportions and facial balance.

**Table 1:** Cephalometric values Changes such as "↑ Then ↓" indicate an intermediate variation; however, the net change was calculated between the initial and post-surgical values. The arrows (↑ / ↓) indicate whether the measurement increased or decreased between those two specific time points. IMPA: Incisor mandibular plane angle; ANB: A-nasion-B

Cephalometric Measurement	Normal Values	Initial Values	Pre-surgical Values	Post-surgical Values	Change (Initial → Post)
SNA (°)	82 ± 2	75.9	80.2	88.3	↑ Increased
SNB (°)	80 ± 2	77.1	78.9	84.7	↑ Increased
ANB (°)	2 ± 2	-1.2	-1.3	3.6	↑ Increased
Wits (mm)	0 mm (±1 for males, ±2 for females)	-3.0	-2.0	-4.0	↓ Decreased
Maxillary Depth (°)	90 ± 3	100.0	100.7	108.0	↑ Increased
Mandibular Depth (°)	87 ± 3	98.8	101.6	104.1	↑ Increased
Lower Facial Height (°)	47% of total facial height	39.3	40.9	42.3	↑ Increased
Facial Axis (°)	90–93	79.4	82.8	92.4	↑ Increased
Upper Incisor Inclination (°)	22 ± 6	103.3	105.5	112.3	↑ Increased
Linear INA (mm)	4 ± 2	7.7	4.3	5.5	↓ Decreased (then ↑)
IMPA (°)	90 ± 5	85.4	89.6	86.1	↑ Then ↓ (overall +0.7)
Linear INB (mm)	4 ± 2	4.0	4.1	7.5	↑ Increased
Horizontal Overjet (mm)	2–4	1.2	1.8	3.5	↑ Increased
Interincisal Angle (°)	135 ± 10	121.0	109.1	123.3	↑ Decreased then ↑

Dental changes included an increase in upper incisor inclination and linear INB, while incisor mandibular plane angle (IMPA) showed minor overall change. Linear INA decreased initially but partially recovered post-surgery. Functional occlusion improved, with horizontal overjet increasing to the normal range and the interincisal angle returning closer to normal values after initial reduction. Overall, the cephalometric measurements reflect significant skeletal and dental improvements following treatment.

Transverse diagnosis

The origin of the facial asymmetry was carefully assessed with CBCT, revealing contributions from the cranial base, both the maxilla and the mandible, as well as a rotation of the cervical vertebrae.

**Figure 4:**
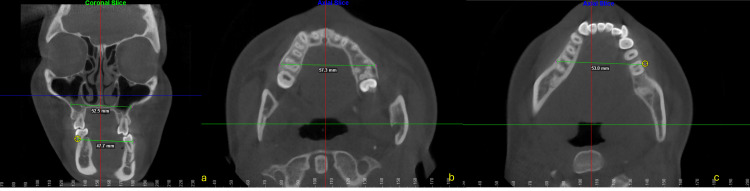
Tomographic slices in different planes obtained using cone-beam computed tomography (CBCT). a. Coronal slice: The relationship between the upper dental roots and adjacent anatomical structures is shown. Transverse measurements of the maxilla at the level of the first molars are highlighted, with distances. b. Axial slice (maxilla): The upper arch is visualized in the axial plane, showing an interpremolar/intermolar transverse distance. c. Axial slice (mandible): The lower arch is observed in the axial plane as well, with a transverse measurement of 53.0 mm between the lower molars.

When CBCT images were subjected to a transverse analysis using the 3D Slicer software (http://www.slicer.org), the mean mandibular width and breadth were found to be 64.5 mm and 57.0 mm, respectively, and a maxillomandibular discrepancy of 3 mm was diagnosed using Pennsylvania analysis. The intercanine and maxillary intermolar distances were 34 and 33 mm, respectively. Measurements of the mandible revealed an intermolar width of 44 mm and an intercanine distance of 24 mm (Figure 4).

The details of the cephalometric changes are shown in Table 1. From the initial to the post-surgical assessment, the patient showed notable improvements in maxillomandibular relationships and dental inclinations. The SNA, SNB, and ANB angles increased, reflecting advancement of both the maxilla and mandible and improved sagittal jaw relationships. Maxillary and mandibular depths also increased, indicating forward positioning of both jaws. Lower facial height and facial axis increased, suggesting enhanced vertical proportions and facial balance.

Dental changes included an increase in upper incisor inclination and linear INB, while IMPA showed minor overall change. Linear INA decreased initially but partially recovered post-surgery. Functional occlusion improved, with horizontal overjet increasing to the normal range and the interincisal angle returning closer to normal values after initial reduction. Overall, the cephalometric measurements reflect significant skeletal and dental improvements following treatment.

Vertical diagnosis

**Figure 5:**
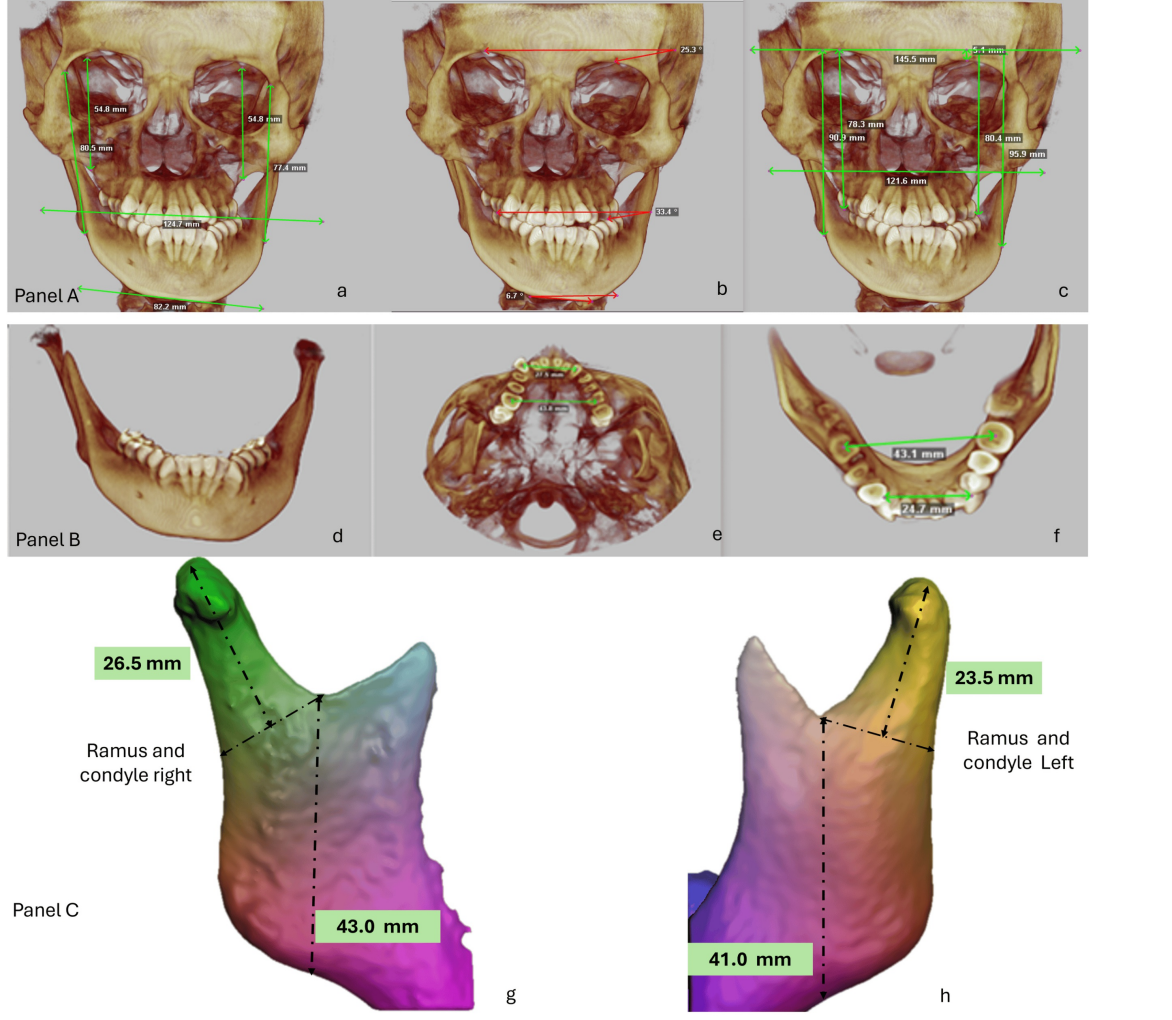
Three-dimensional reconstructions obtained from computed tomography for craniofacial and mandibular morphometric analysis. Panel A (a–c): Frontal views of the skull showing key craniofacial measurements (in millimeters), including orbital height and width, zygomatic distance, and mandibular symmetry. Panel B (d–f): Views of the mandibular complex from different angles: inferior (d), transverse (e), and oblique (f), with specific measurements such as mandibular width and interdental distances. panel C: 3D models of the right (left image) and left (right image) mandibular ramus, displaying measured heights and lengths from anatomical landmarks (values in mm). Image Credits: Dr. Gerardo Martínez-Suárez

The 3D vertical analysis revealed an orbital height disparity of 5.5 mm, with the left side positioned lower. The OP exhibited a 4 mm tilt and was inclined at 33° in relation to a horizontal reference line. The bottom left margin of the chin was situated 6.5 mm inferiorly. The right mandibular ramus measured 43 mm, compared to 41.0 mm on the left side. Condylar height was 26.5 mm on the right and 23.2 mm on the left, indicating mild skeletal asymmetry (Figure 5).

Table 2 presents cephalometric measurements of the mandible before and after surgery, highlighting changes in the length and height of the mandibular bodies and rami, as well as in condylar length and volume. The right mandibular body decreased by 6 mm, while the left increased by 4 mm, indicating correction of the asymmetry. The ramus heights and condylar lengths showed slight reductions on both sides, whereas the condylar volume remained almost constant. Overall, these changes reflect improved mandibular alignment and symmetry following the surgical intervention.

**Table 2:** Linear and volumetric dimensions of the body, ramus and condyle between pre-treatment vs post-treatment

Cephalometric Measurement	Initial Values	Post-surgical Values	Δ (Change)	Trend
Right body length (mm)	83	78	-5	↓
Left body length (mm)	75	78	+3	↑
Right ramus height (mm)	43	42.3	-0.7	↓
Left ramus height (mm)	41	40.5	-0.5	↓
Right condylar length (cm)	26.5	25.4	-1.1	↓
Left condylar length (cm)	23.2	22.9	-0.3	↓
Right condylar volume (cm³)	1.5	1.5	0	→
Left condylar volume (cm³)	1.5	1.4	-0.1	↓

Upper airways 3D assessment

Upper airway segmentation was carried out in 3D Slicer using the “CT-Air” visualization preset. A region of interest (ROI) was then created with the Crop Volume extension, and this ROI was used to delineate the upper airway for segmentation.

Anatomical Boundaries

In the sagittal view, the anatomical limits of the ROI were established using precise reference planes. The superior boundary was defined by a plane passing below the body of the sphenoid bone, just above the inferior margin of the sphenoid sinus. The inferior boundary corresponded to a plane at the level of the lower mandibular border, ensuring complete inclusion of the mandibular contour without encompassing cervical structures. The posterior boundary was set using a plane passing through the center of the basilar part of the occipital bone, coinciding with the cephalometric landmark basion. Finally, the anterior boundary was determined by a plane intersecting the most posterior point of the palatal plane, coinciding with the cephalometric landmark posterior nasal spine (PNS). These boundaries ensure standardization of orientation and comparability of three-dimensional measurements across all subjects analyzed.

Following the same semi-automatic segmentation method, a median smoothing filter of 0.75 mm was applied, followed by Gaussian smoothing of 1.00 mm, maintaining the original scale 1:1 and the RAS coordinate system. The surfaces were exported in STL (stereolithography) format.

**Figure 6:**
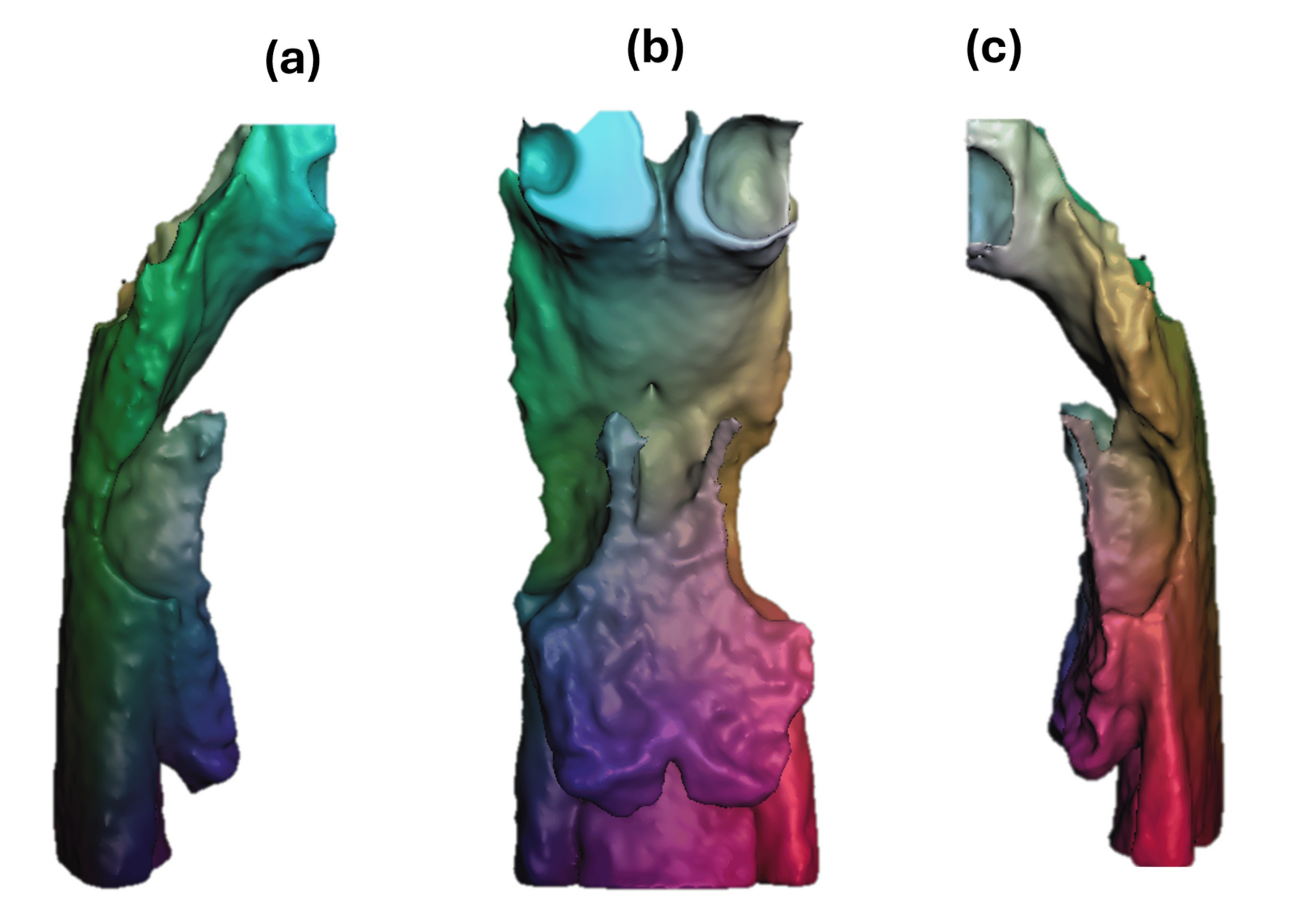
Multiview 3D visualization of the upper pharyngeal airway (UPA). This panel presents three anatomical views of the upper pharyngeal airway (UPA) in its 3D reconstruction: Right lateral view (a): shows the sagittal contour of the airway, allowing clear visualization of the anteroposterior dimension and potential areas of narrowing in the oropharyngeal and hypopharyngeal regions.  Frontal view (b: provides a direct anterior perspective of the airway lumen, highlighting asymmetries or lateral wall collapses. Left lateral view (c): complements the right view, offering a comprehensive bilateral assessment of airway shape and patency. Image Credits: Dr. Gerardo Martínez-Suárez

At the beginning of treatment, the upper pharyngeal airway (UPA) presented a reduced shape and volume. In certain regions-particularly within the mid- and lower-pharyngeal areas-noticeable narrowings or partial collapses of the airway lumen were observed, suggesting increased obstruction or reduced airway patency. The total measured airway volume was 21.5 cm³. This 3D reconstruction illustrates the UPA prior to treatment, highlighting marked constrictions, especially in the oropharyngeal and hypopharyngeal regions, consistent with diminished airway patency (Figure 6).

Treatment objectives

Maxilla

Intrude the right side by 3 mm and descend the left side by 2 mm to level the occlusal plane, advance the maxilla by 5 mm to correct the paranasal depression and improve sagittal coordination with the mandible, enhance vertical exposure of the upper incisors, and provide better support to the upper lip (lip taper).

Mandible

Rotate the mandible counterclockwise to improve chin projection, eliminate skeletal asymmetry, reduce lower facial height, correct retrognathism and Class II malocclusion, highlighting modifications in the mandibular body length, rotation to the right a 7 mm setback on the right side, and 5 mm advancement of the left mandibular body toward the right and center and level the inferior border of the chin.

Treatment alternatives

Dental Compensation

An orthodontic therapy was recommended that does not involve orthognathic surgery, entailing the extraction of teeth 15 and 25 to align the upper midline with the facial midline, and the extraction of teeth 34 and 44 to rectify the Class III molar connection on the right side. A mini-implant was to be inserted into the mandibular shelf to distalize quadrant IV and align the arches. Nonetheless, the patient's parents did not consent to this treatment choice.

Dental Decompensation

A combined orthodontic and orthognathic surgical intervention was selected to accomplish dental decompensation, leveling, aligning, and coordinating the dental arches. Extractions of teeth 14 and 24 were warranted to align the upper midline with the facial midline, while teeth 35 and 45 were removed to enable dental decompensation and generate room for rectifying the pronounced lingual inclination of the teeth in quadrant III. Soft tissues naturally attempt to mask the underlying skeletal discrepancy through varying degrees of dental proclination. In the anteroposterior plane, the teeth tend to incline toward one another in an effort to reduce the skeletal disharmony.

Pre-surgical orthodontic preparation

Soft tissues naturally attempt to mask the underlying skeletal discrepancy through varying degrees of dental proclination. In the anteroposterior plane, the teeth tend to incline toward one another to reduce the skeletal disharmony.

Pre-surgical orthodontic preparation was conducted utilizing Roth 0.022 x 0.025 Ovation® appliances (GAC). A transpalatal arch was implemented to secure molar anchoring during the retraction of the front maxillary segment. After extracting the upper first premolars and lower second premolars, the orthodontic team executed leveling, alignment, and second- and third-order motions seamlessly and effectively, which facilitated the correction of both the Curve of Spee and the Curve of Wilson while establishing arch coordination. During the alignment and leveling phase, appropriate arch shape and coordination were established. Closure of space in the maxillary arch was accomplished utilizing a 0.017 x 0.025-inch double key loop (DKL) stainless steel archwire.

In the subsequent phase, anchoring loss was intentionally provoked by employing a stainless steel archwire of identical size (0.017 x 0.025 inches), which was selectively reduced in thickness along its edges to promote movement. Post-operative orthodontic preparation spanned 18 months and was finalized utilizing 0.021 x 0.025-inch stainless steel archwires.

Treatment progress

Left profile views, as well as frontal views at rest and smiling, allow for the assessment of changes in facial profile and aesthetic expression. The intraoral images show dental alignment in both arches with fixed orthodontic appliances in place. Upper and lower occlusal views provide insight into the progress of dental inclination decompensation, alignment, and arch coordination-essential elements for achieving correct maxillomandibular basal positioning during surgery.

**Figure 7:**
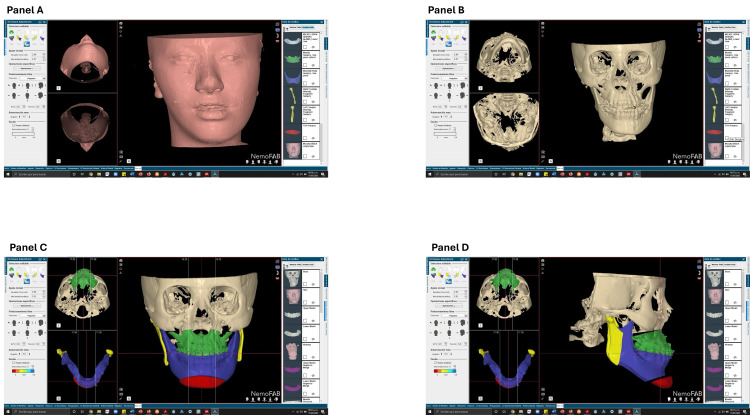
Virtual planning of orthognathic treatment Virtual surgical planning using Face-Airway-Bite (FAB)®. Panel A: 3D reconstruction of facial soft tissue. Panel B: Bone segmentation based on tomographic images. Panel C: Virtual delineation of color-coded maxillofacial segments (Green: Maxilla, Blue: Mandibular Body, Red: Chin, Yellow: Mandibular Ramus). Panel D: Three-dimensional simulation of the planned surgical movements.

Preoperative documentation, surgical forecasting, and model surgery were conducted in accordance with the established planification (Figure 7). 

Table 3 presents a summary of the cephalometric changes. The cephalometric analysis revealed several significant improvements from the initial to the post-surgical stage. The convexity angle increased markedly (+6.5°), reflecting enhanced facial projection and profile harmony. A substantial improvement was also observed in the nasolabial angle, which increased by 18.5°, moving from a reduced value to within the normative range, indicating a more balanced soft-tissue relationship.

The mentocervical distance showed a notable increase (+9.5 mm), suggesting improved chin-neck definition. Additionally, the lower lip position shifted by −3.0 mm toward a more ideal position relative to the E-line. The chin position advanced by 4.0 mm, approaching normative values and contributing to improved lower facial balance. A significant correction was also observed in the occlusal plane-SN angle, which decreased by 15°, aligning more closely with the expected physiological range.

Conversely, upper incisor exposure, interlabial distance, and upper lip position exhibited no clinically relevant changes during the course of treatment.

**Table 3:** Cephalometric summary of facial profile Patterns like "↑ then ↓" represent a temporary fluctuation; however, the overall change was determined based on the difference between the pre-surgical and post-surgical values. The arrows (↑ / ↓) denote whether the measurement rose or fell between those specific time points.

Cephalometric Measurement	Normal Values	Initial	Pre-surgical	Post-surgical	Δ Initial → Post	Change
Convexity Angle (°)	13 ± 3	12	18	18.5	6.5	↑ Increased
Nasolabial Angle (°)	102 ± 8	88.5	118.5	107	18.5	↑ Increased
Upper Incisor Exposure (mm)	+2 to +4 mm	+2.0	4	2	0	→ No change
Interlabial Distance (mm)	1–4 mm	0	6	0	0	→ No change
Mentocervical Distance (mm)	~42–50 mm (variable)	36.5	42.5	46	9.5	↑ Increased
Upper Lip Position (mm)	0 to -4 mm (vs E-line)	3	1	3	0	→ No change
Lower Lip Position (mm)	0 to -2 mm (vs E-line)	3	5	0	-3	↓ Decreased
Chin Position (mm)	0 to -4 mm (vs N-Pg)	-5	-5.5	-1	4	↑ Increased
Occlusal Plane–SN Angle (°)	14–16°	31	8.1	16	-15	↓ Decreased

Digital surgical planning

**Figure 8:**
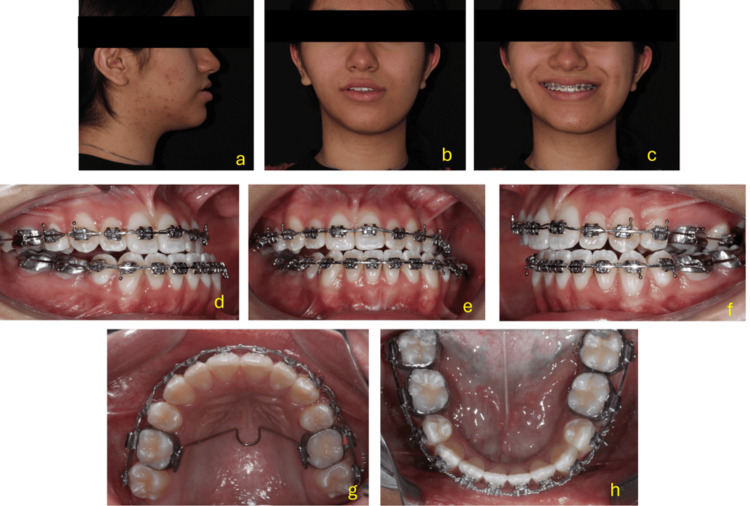
Extraoral and intraoral clinical photographs taken during the dental decompensation phase prior to orthognathic surgery. (a–c): Extraoral photographs showing the patient in profile view (a), at rest (b), and smiling (c). (d–f): Intraoral frontal (e), right (d), and left (f) lateral views displaying the alignment and position of the dental arches with fixed orthodontic appliances in place. (g–h): Occlusal views of the upper (g) and lower (h) arches showing arch form, bracket positioning, and archwire progression.

For the digital surgical planning, a CBCT scan was requested with a 15 × 15 cm field of view (FOV) and a voxel size of 0.3 × 0.3 × 0.3 mm, using the NemoTec digital software. In the lateral and PNC views, a line was traced 7 mm from the glabella point (frontal reference plane). In the frontal view, the bicigomatic line, facial midline, intercanine line, and the inferior border of the chin were traced (Figure 8).

Bone scintigraphy study

To rule out condylar hyperplasia prior to orthognathic surgery, a bone scintigraphy study was requested using the SPECT technique (Single Photon Emission Computed Tomography), which provides three-dimensional images in axial, sagittal, and coronal planes. This technique has been proven to be more sensitive compared to planar imaging.

In this diagnostic test, a radioactive substance known as a radiopharmaceutical (99mTc-MDP) is administered intravenously. Over the course of three hours, it biodistributes into the hydroxyapatite. Images are then acquired, and the percentage of radiotracer uptake in the mandibular condyles is measured. A 10% difference in uptake between the condyles is indicative of asymmetry, and uptake greater than 55% suggests condylar hyperplasia.

**Figure 9:**
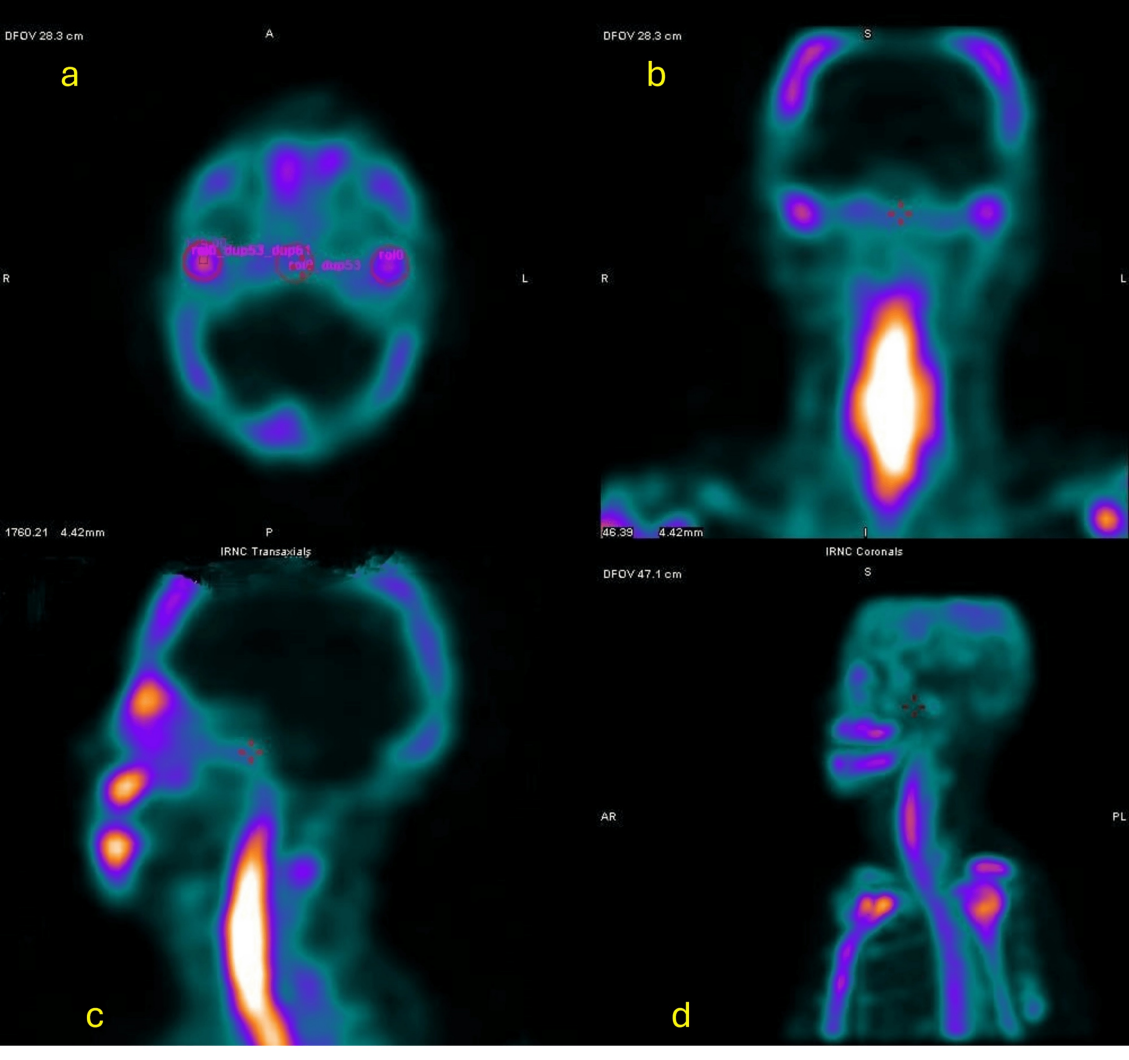
Single photon emission computed tomography (SPECT) imaging of the craniofacial region for functional evaluation of the temporomandibular joints (TMJs). (a) Axial view showing bilateral uptake in the TMJs, indicating active bone remodeling or inflammation. (b) Coronal view confirming symmetrical tracer uptake localized to both TMJs. (c) Sagittal view displaying tracer distribution along the mandibular ramus and cervical region. (d) 3D reconstruction in lateral view, visualizing functional activity in the TMJs and cervical spine. Areas of high uptake are visualized in bright white/yellow, indicating increased metabolic activity.

The SPECT images, in coronal, axial, and transverse sections, revealed the following findings: In the axial view, regions of interest (ROIs) were drawn on both mandibular condyles and the clivus. The radiotracer uptake was 48.8% in the right condyle and 51.2% in the left condyle, indicating a minimal difference between sides. These results allowed the exclusion of active condylar hyperplasia, as shown in Figure 9.

Surgical technique

Presurgical orthodontic decompensation was carried out to align and level the teeth while correcting dental compensations, facilitating accurate skeletal repositioning during surgery. Occlusal splints were used intraoperatively to guide the maxillomandibular complex into the planned surgical position and ensure proper postoperative occlusal relationships.

Bimaxillary orthognathic surgery was performed using intermediate and final splints to guide the maxillomandibular complex during surgery. Le Fort I osteotomies were used to correct the sagittal maxillary deficiency and the cant of the maxillary occlusal plane. The intermediate splint was employed to position the maxilla temporarily relative to the mandible before final adjustment, while the final splint ensured the definitive skeletal alignment prior to fixation. A 5 mm maxillary advancement was planned to address the depression in the paranasal region, combined with an asymmetric adjustment of the occlusal plane: 3 mm intrusion on the right and 2 mm extrusion on the left.

Correction of mandibular asymmetry was achieved through an asymmetric setback using right- and left-sided sagittal split ramus osteotomies, including a 5.5° rotation to the right, a 7 mm setback on the right, and a 5 mm advancement of the left mandibular body toward the right. These movements were intended to center the mandible and correct the skeletal Class III relationship. The inferior borders of the chin were reshaped by reducing the bony prominence. Final positioning followed the preoperative surgical plan to ensure facial symmetry and proper alignment of the lip commissures.

Semi-rigid fixation was applied to the maxilla, rigid fixation to the right mandibular side, and two bicortical screws to the left mandibular side. Twelve weeks post-surgery, a stainless-steel arch with a reinforced vertical loop was placed on the maxilla to close the residual gap between teeth 22 and 23. On the mandible, a 0.017 x 0.025-inch stainless-steel arch with an elastic chain from tooth 7 to 7 was used to close residual spaces. Vertical and Class III intermaxillary elastics were applied to achieve the final occlusion. Nine months were required postoperatively to complete space closure and finalize the detailing phase.

Treatment results

The correction of facial asymmetry was achieved through leveling of the maxillary plane, a 7 mm setback on the right side, a 5 mm advancement of the left mandibular body toward the right, and a 5.5° rotation of the mandibular body toward the right side.

**Figure 10:**
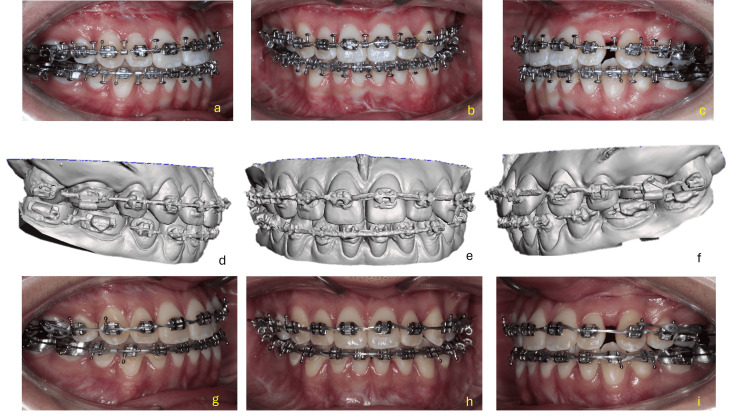
Intraoral clinical photographs taken after orthognathic surgery. Top row (a–c): Intraoral photographs of the patient showing the results six weeks after orthognathic surgery. Middle row (d–f): Right lateral (d), frontal (e), and left lateral (f) intraoral views obtained using an intraoral scanner, showing the alignment and positioning of the dental arches with fixed orthodontic appliances. Bottom row (g–i): Right lateral (g), frontal (h), and left lateral (i) intraoral views illustrating the finishing phase, bracket positioning, and treatment progress.

The post-treatment images exhibit a significant enhancement in facial aesthetics, especially in the profile and the rectification of asymmetry in the lower third of the face. Intraoral photos demonstrate substantial alterations in dental relationships, including the leveling of the occlusal plane and the establishment of bilateral Class I molar and canine relationships. The lower midline presents a minor deviation to the left, and the configuration of the dental arch is acceptable. Cephalometric alterations encompass an elevation in the ANB angle from -1.2° to 4°, signifying an enhanced maxillomandibular connection. The angle between the mandibular incisor and the mandibular plane increased from 85° to 89°. The upper incisors were positioned upright by means of space closure and retraction of the anterior maxillary segment, enhancing upper lip support and aiding in maxillary advancement. The visibility of the top incisors is now symmetrical, enhancing a more balanced and straight facial profile with adequate chin projection. The nasolabial angle was enhanced, and the interlabial gap diminished. The ultimate occlusion was attained with bilateral Class I molar and canine connections. Both horizontal and vertical overbites were diminished to 2 mm, achieving ideal functionality and aesthetics. The maxillary and mandibular dental midlines were almost aligned with the facial midline (Figure 10).

**Figure 11:**
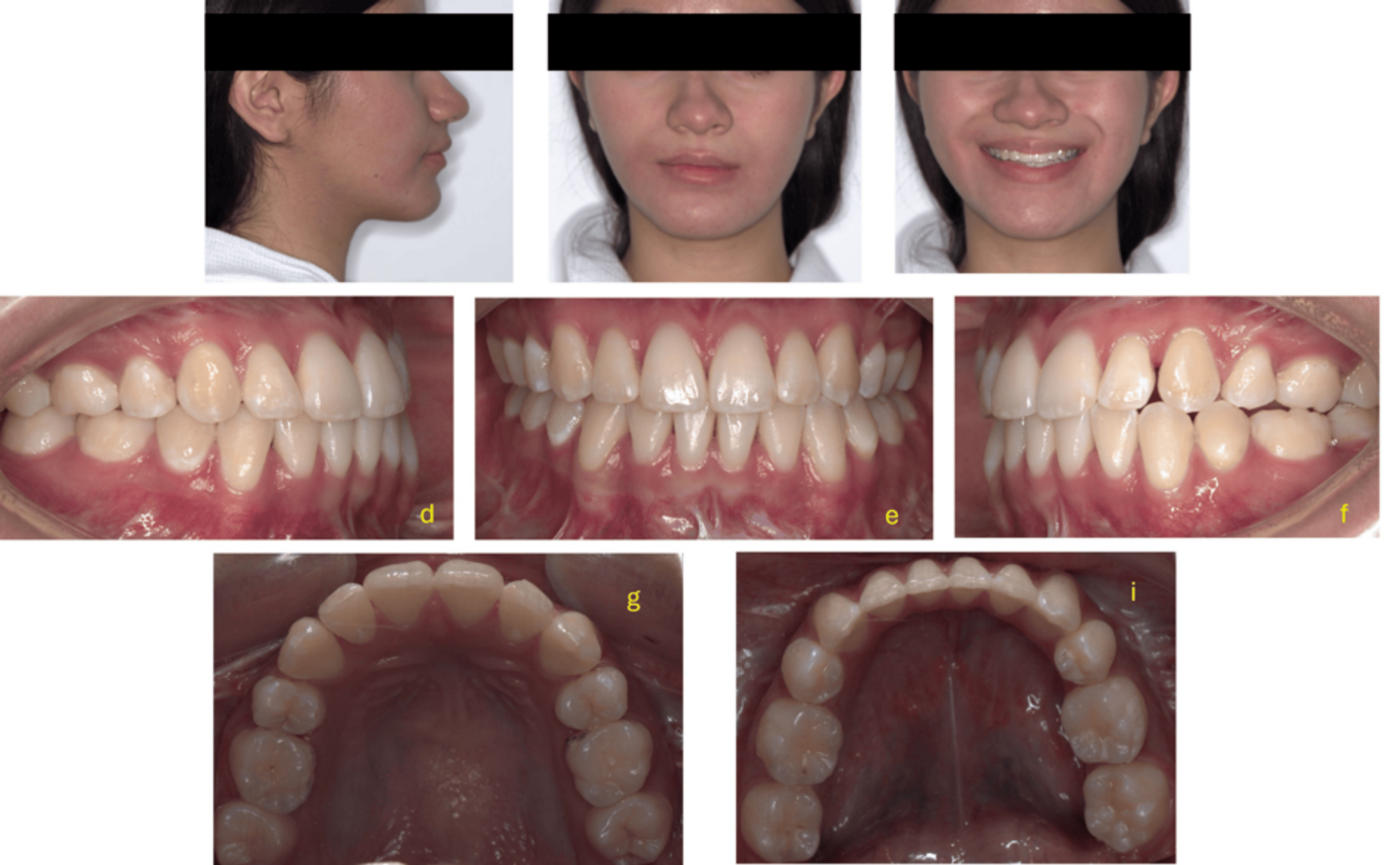
Extraoral and intraoral clinical photographs taken at the end of treatment. Top row (a–c): Extraoral photographs of the patient showing the final results after orthognathic surgery. Middle row (d–f): Right lateral (d), frontal (e), and left lateral (f) intraoral views showing the final dental alignment and occlusion. Bottom row (g, h): Upper occlusal (g) and lower occlusal (h) views illustrating the arch form and the finishing phase of treatment.

Figure 11 displays the postoperative extraoral and intraoral clinical records. Right and left profile views, frontal views at rest and smiling are presented for facial aesthetic analysis. The second row shows intraoral images in occlusion: right lateral, frontal, and left lateral views, demonstrating sagittal (Class I) and transverse relationships of the dental arches. The maxillary and mandibular occlusal views allow evaluation of arch form and dental alignment. These records constitute a fundamental part of the results obtained from comprehensive orthodontic-surgical treatment planning.

**Figure 12:**
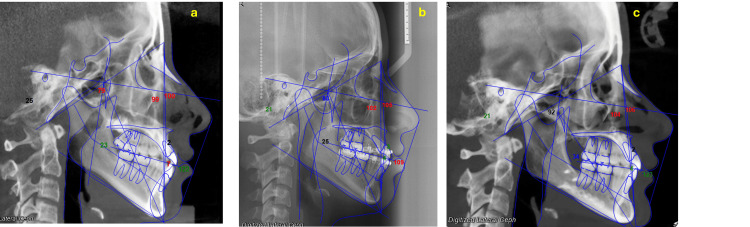
Radiographic comparison pre-treatment and post-treatment Radiographic comparison in orthodontics and orthognathic surgery: (a) Pretreatment radiograph showing malocclusion and initial skeletal discrepancy; (b) Pre-surgical orthodontic preparation radiograph with aligned and decompensated teeth; (c) Post-orthognathic surgery radiograph showing skeletal correction and final occlusion. Cephalometric tracings illustrate changes in key cephalometric values across the treatment stages.

The final cephalometric tracing illustrate the changes achieved following orthodontic treatment and orthognathic surgery. The pretreatment radiograph shows the malocclusion and initial skeletal discrepancy, while the pre-surgical orthodontic preparation radiograph demonstrates the alignment and decompensation of the teeth. The post-orthognathic surgery radiograph reveals the skeletal correction and the establishment of the final occlusion. Overall, the cephalometric tracings highlight the progressive modifications in key cephalometric measurements throughout the different stages of treatment (Figure 12).

CMF registration and ICP alignment

Craniofacial model alignment was performed using the CMF Registration module in 3D Slicer, which allows for surface-based registration of 3D anatomical models. For this study, the pre-surgical maxilla was designated as the fixed model, and the post-surgical maxilla served as the moving model. Registration was conducted using the iterative closest point (ICP) algorithm. Parameters were set to a maximum of 100-200 iterations, a tolerance of 0.001-0.01 mm, and optional landmark-based initialization was applied to improve convergence.

After completion of the ICP procedure, the registration was verified by inspecting the overlap of the models in the 3D view and confirming alignment across the axial, sagittal, and coronal planes. Minor discrepancies were corrected through manual landmark refinement when necessary, ensuring accurate superimposition for subsequent quantitative analysis.

Figure 11 superimposition illustrates areas of surface discrepancy between the two time points, highlighting position changes in the condylar region after surgical intervention. A CBCT scan was requested with a 15 × 15 cm field of view (FOV) and a voxel size of 0.3 × 0.3 × 0.3 mm, using 3D Slicer software. All segmentations and superimpositions were performed. Initial (beige) and final (green) scans, allowing observation of changes in the position of the maxilla and mandible after bimaxillary orthognathic surgery. Bottom row: Post-surgical model, focusing especially on the mandible, facial symmetry, and intermaxillary relationship. These were generated by automatic alignment using 3D Slicer with the surface registration extension for analysis of changes.

**Figure 13:**
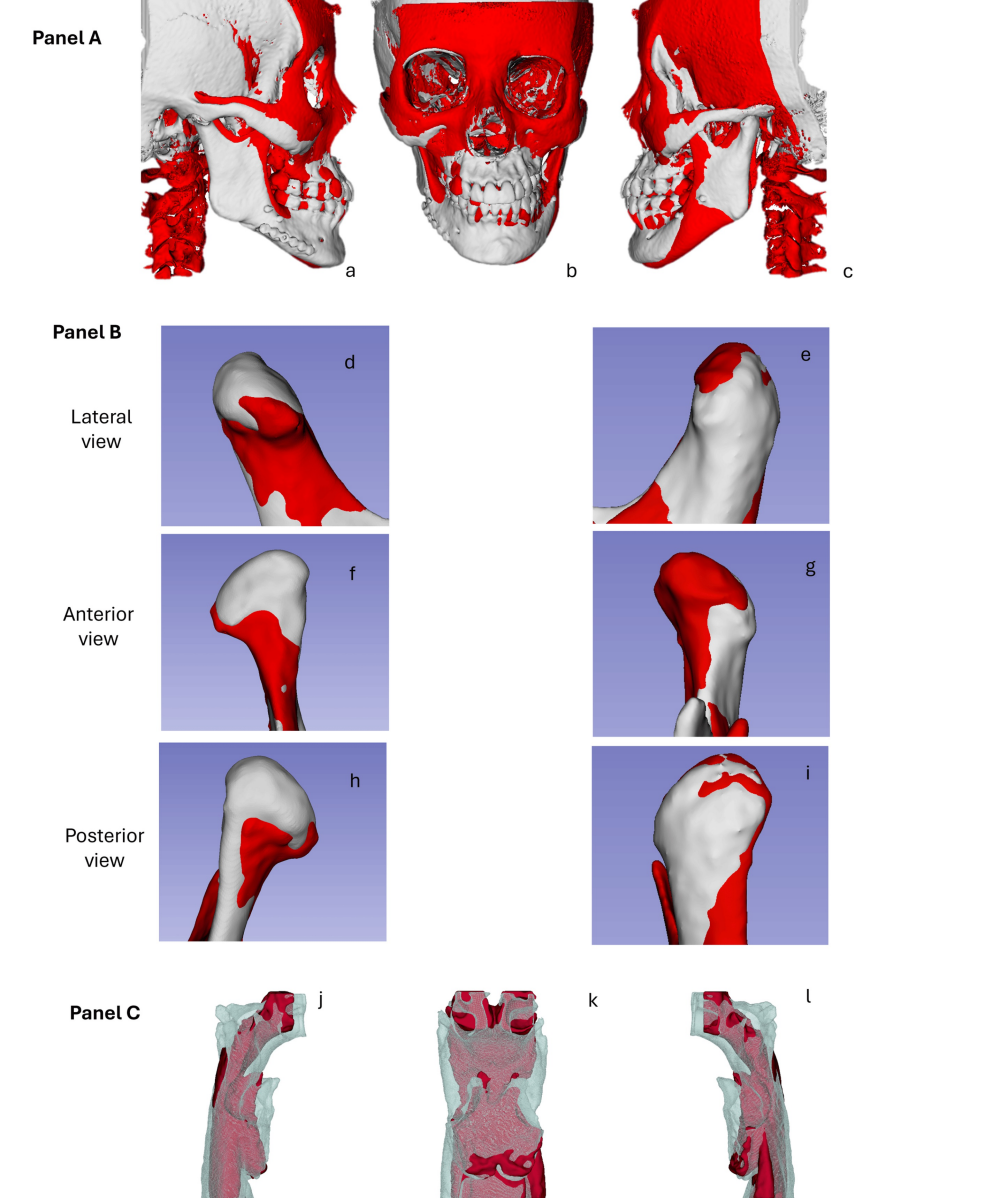
Three-dimensional superimposition of mandibular condyles in different views The red surface represents the pre-treatment (fixed) model, while the white surface corresponds to the post-treatment (moving) model. Each column displays the condylar region from various anatomical perspectives, enabling visual assessment of morphological and positional changes. The superimposition illustrates areas of surface discrepancy between the two time points, highlighting position changes in the condylar region after surgical intervention. All segmentations and superimpositions were performed using 3D Slicer software. Image Credits: Dr. Gerardo Martínez-Suárez

This 3D visualization illustrates the morphological changes in the upper pharyngeal airway (UPA) before and after treatment. In the pretreatment model (red), the airway appears more constricted, particularly in the mid and lower pharyngeal regions, with evident areas of narrowing and partial airway collapse. These features are suggestive of reduced airway patency and potential airflow obstruction. In contrast, the post-treatment model (beige/translucent) shows a clear increase in airway volume and expansion of the pharyngeal walls, particularly in the lateral and anteroposterior dimensions. This suggests an improvement in airway patency and a likely reduction in obstruction risk. Overall, the comparison demonstrates a notable enlargement and reshaping of the airway following treatment, supporting the effectiveness of the intervention in improving upper airway morphology (Figure 13).

## Discussion

A comprehensive diagnostic approach is essential in patients with Class III malocclusion and facial asymmetry, as these cases often involve complex skeletal, dental, and functional discrepancies. Individualized evaluation of the temporomandibular joint (TMJ) using cone-beam computed tomography (CBCT) is critical to assess joint morphology, condylar position, and potential asymmetries that may contribute to the malocclusion. Furthermore, in cases where active condylar hyperplasia is suspected, it is imperative to complement the structural assessment with functional imaging such as Single Photon Emission Computed Tomography (SPECT), which offers higher sensitivity in detecting metabolic activity in the condyles. This integrative diagnostic process ensures accurate treatment planning and helps prevent relapse or progressive asymmetry post-treatment.

The combined orthodontic and surgical treatment led to significant improvements in both facial aesthetics and occlusal function. The correction of lower third facial asymmetry was achieved through improved chin projection and mandibular positioning. Bilateral Class I occlusion, alignment of dental midlines with the facial midline, and leveling of the occlusal plane were accomplished, with enhancements in lip support, incisor visibility, and nasolabial angle-resulting in a more balanced facial profile and functional bite.

Cephalometric evaluation revealed highly favorable skeletal and dental changes following orthodontic-surgical treatment. The substantial increases in SNA and SNB values demonstrated successful three-dimensional repositioning of both the maxilla and mandible, which transformed an initial Class III skeletal relationship (initial ANB −1.2°) into a stable Class I relationship (final ANB 3.6°). The correction of the facial axis, which improved from 79.4° to 92.4°, reflects a significant enhancement in facial growth direction, resulting in a more balanced and functional craniofacial structure. Likewise, the increase in overjet-from 1.2 mm initially to 3.5 mm post-surgery-and the normalization of the interincisal angle, which improved from 121° to 123.3°, indicate a more harmonious and functional dental relationship. Additionally, the increase in lower facial height, from 39.3° to 42.3°, contributed to a more proportionate facial profile. Collectively, these changes demonstrate comprehensive improvements in facial structure, occlusion, and function, fully consistent with the objectives of orthodontic-surgical treatment.

Digital planning and 3D imaging played a critical role in accurately diagnosing skeletal discrepancies and customizing treatment, particularly in repositioning the maxilla and mandible to optimize function and aesthetics [14-17].

Ayub et al. [18] ​​​emphasize that the successful management of skeletal Class III malocclusion via orthognathic surgery relies heavily on strong interdisciplinary collaboration. In the present case, the treatment sequence was strategically designed based on a comprehensive diagnostic evaluation conducted jointly by the orthodontist and the maxillofacial surgeon, prioritizing the assessment of skeletal imbalances and occlusal relationships.

Skeletal stability following surgery is another potentially clinically significant outcome that may depend on the fixation tool selection, including monocortical and bicortical fixation [12,19]. In this case, rigid fixation was applied to the mandible and semi-rigid fixation to the maxilla, which provided excellent stability in both the intermaxillary relationship and condylar position. A notable improvement in mandibular dynamics was also observed, with more symmetrical movements during mouth opening and lateral excursions. Bimaxillary orthognathic surgery continues to stand out, as rigid internal fixation offers greater postoperative stability compared with isolated mandibular procedures, reducing the risk of relapse and promoting more predictable and long-lasting outcomes.

Bimaxillary orthognathic surgery significantly alters the face, airway, and bite. In fact, aesthetics is one of the main motivations for patients to undergo this type of surgery [14,16,20]. Bimaxillary orthognathic surgery can impact the airway, with mixed results. Some patients experience improved breathing post-surgery [20].

## Conclusions

Facial asymmetries pose a significant challenge in orthodontic-surgical treatment, making a thorough clinical, morphometric, and cephalometric evaluation indispensable from the beginning.

The successful management of this clinical case represented a significant challenge due to the severity of the skeletal, dental, and soft tissue asymmetry. Early detection of these conditions allowed for more precise treatment planning and improved predictability through a decompensatory orthodontic treatment prior to performing the planned surgical procedures. Additionally, assessing condylar growth-particularly in cases of suspected asymmetric condylar hyperplasia-is essential to ensure stable medium- and long-term outcomes, especially in younger patients.

The comprehensive and multidisciplinary management of this case throughout all treatment phases was fundamental to achieving the initial objectives. The pre-surgical stage included a thorough evaluation of all diagnostic elements, consisting of detailed clinical assessments, diagnostic imaging, dental model mounting, and cephalometric analysis to guide treatment planning. The surgical phase involved virtual planning of bimaxillary orthognathic surgery, executed with meticulous intraoperative detail and control to achieve the planned skeletal and occlusal corrections. Postoperatively, close follow-up focused on final detailing, occlusal adjustments, monitoring skeletal stability, and evaluating functional and esthetic outcomes.
